# Tahyna virus genetics, infectivity, and immunogenicity in mice and monkeys

**DOI:** 10.1186/1743-422X-8-135

**Published:** 2011-03-24

**Authors:** Richard S Bennett, Anthony K Gresko, Brian R Murphy, Stephen S Whitehead

**Affiliations:** 1 Laboratory of Infectious Diseases, National Institute of Allergy and Infectious Diseases, National Institutes of Health, Bethesda, MD 20892, USA

## Abstract

**Background:**

Tahyna virus (TAHV) is a human pathogen of the California encephalitis virus (CEV) serogroup (*Bunyaviridae*) endemic to Europe, Asia, and Africa. TAHV maintains an enzootic life cycle with several species of mosquito vectors and hares, rabbits, hedgehogs, and rodents serving as small mammal amplifying hosts. Human TAHV infection occurs in summer and early fall with symptoms of fever, headache, malaise, conjunctivitis, pharyngitis, and nausea. TAHV disease can progress to CNS involvement, although unlike related La Crosse virus (LACV), fatalities have not been reported. Human infections are frequent with neutralizing antibodies present in 60-80% of the elderly population in endemic areas.

**Results:**

In order to determine the genomic sequence of wild-type TAHV, we chose three TAHV isolates collected over a 26-year period from mosquitoes. Here we present the first complete sequence of the TAHV S, M, and L segments. The three TAHV isolates maintained a highly conserved genome with both nucleotide and amino acid sequence identity greater than 99%. In order to determine the extent of genetic relatedness to other members of the CEV serogroup, we compared protein sequences of TAHV with LACV, Snowshoe Hare virus (SSHV), Jamestown Canyon virus (JCV), and Inkoo virus (INKV). By amino acid comparison, TAHV was most similar to SSHV followed by LACV, JCV, and INKV. The sequence of the G_N _protein is most conserved followed by L, N, G_C_, NS_S_, and NS_M_. In a weanling Swiss Webster mouse model, all three TAHV isolates were uniformly neurovirulent, but only one virus was neuroinvasive. In rhesus monkeys, the virus was highly immunogenic even in the absence of viremia. Cross neutralization studies utilizing monkey immune serum demonstrated that TAHV is antigenically distinct from North American viruses LACV and JCV.

**Conclusions:**

Here we report the first complete sequence of TAHV and present genetic analysis of new-world viruses, LACV, SSHV, and JCV with old-world viruses, TAHV and INKV. Using immune serum generated in monkeys against TAHV, LACV, and JCV, we have demonstrated cross-neutralization within the CEV serogroup. Such cross reactivity may complicate virus identification, especially following JCV infection which elicited antibodies that cross neutralized both LACV and TAHV. These data also suggest that a single vaccine could generate a cross-neutralizing antibody response which may provide protection against CEV serogroup viruses from a wide geographic range.

## Introduction

Tahyna virus (TAHV), family *Bunyaviridae*, is a mosquito-borne pathogen endemic in Europe, Asia and Africa [1-8]. TAHV is a member of the California encephalitis virus (CEV) serogroup and is closely related to La Crosse virus (LACV) found in North America [9-11]. Members of this family have tri-segmented, negative-sense RNA genomes of approximately 13 kb in total length. The three segments are designated by their size, small (S), medium (M), and large (L) and are complexed with nucleoprotein to form three separate nucleocapsids. The 3' and 5' untranslated regions (UTR) are complementary and highly conserved. The S segment encodes two proteins: the nucleoprotein (N) and a non-structural protein (NS_s_) which inhibits transcription via inhibiting host cell RNA polymerase II resulting in decreased interferon (INF) induction [12,13]. The M segment encodes a single polyprotein (M polyprotein) that is post-translationally processed into two surface glycoproteins (G_N _and G_C_) which are the targets of neutralizing antibodies, and a nonstructural protein, NS_M _[14]. The L segment encodes a RNA-dependent RNA polymerase [15,16].

TAHV maintains an enzootic life cycle with multiple culicine and anopheles mosquito species [1,5]. These mosquitoes feed on hares, rabbits, hedgehogs, and rodents, which serve as amplifying hosts [5,17-19]. The virus can overwinter in either virus-infected eggs or live mosquitoes [5]. During late summer and early fall, TAHV causes a non-fatal flu-like illness in humans and is sometimes called "Valtice fever" with symptoms of fever, headache, malaise, nausea, myalgia, and occasionally bronchopneumonia [4,20]. As with the related LACV, TAHV disease occurs mainly in children and can lead to CNS infection. Human infections are frequent with neutralizing antibodies present in 60-80% of the elderly population in endemic areas [4,20]. In the Czechland and Slovakia regions, at least 200 documented cases have been reported since 1963 [5]. It is evident that the majority of TAHV infections are asymptomatic, mild, or unreported.

Although TAHV infections are common, limited research has been done to understand viral genetics or develop appropriate animal models. We have sequenced the complete genome of three TAHV isolates and their biologically-cloned derivatives in order to identify a TAHV nucleotide sequence associated with wildtype phenotypes both in tissue culture and in mouse and monkey models. We also sought to determine the genetic and antigenic relatedness of TAHV with LACV and Jamestown Canyon virus (JCV) by cross-neutralization using rhesus monkey immune serum. These data may be useful in identifying vaccine candidates for protection against this serogroup of viruses.

## Results

### Sequence analysis of viral genomes

Here we report the first complete sequence of three TAHV strains including the large (L) segment (GenBank accession numbers found in Table 1). We first sought to define a complete genome sequence associated with wild-type virulence in mice by both peripheral and intracerebral routes of inoculation, and second, by sequencing multiple isolates, we sought to determine the genetic diversity of TAHV isolated in different countries at different times. All viruses were biologically cloned to generate a genetically homogeneous preparation for sequence analysis.

**Table 1:** Passage history and geographic location of isolation of Tahyna viruses used in this study

Virus	Strain	Site of isolation	Passage history^a^	GenBank accession number
TAHV/58/CZ	Prototype "92" Bardos	Czechoslovakia	MB, C6/36 p1	n/a^b^
TAHV/58/CZ-cl(1)^c^			MB, C6/36 p1, vero p4	HM036208-10
TAHV/58/CZ-cl(2)^c^			MB, C6/36 p1, vero p4	HM036211-13
				
TAHV/68/FR	C14019-29	France	MB p5, C6/36 p1	n/a
TAHV/68/FR-cl ^c^			MB p5, C6/36 p1, vero p4	HM036214-16
				
TAHV/84/CZ	22595-6	Czechoslovakia	MB p22, C6/36 p1	n/a
TAHV/84/CZ-cl^c^			MB p22, C6/36 p1, vero p4	HM036217-19

^a ^Cell/tissue type followed by number of passages. MB = mouse brain, total number of passages unknown

^b ^Not applicable. Sequence not submitted to GenBank.

^c ^Biologically-cloned derivative.

The initial sequence data from TAHV/58/CZ indicated that the original stock vial of this virus was a mixture of two virus populations. Biological cloning was used to generate pure stocks of both the major and minor population for sequence analysis and phenotyping. Genetic comparison of the major population TAHV/58/CZ-cl(2) and the minor population TAHV/58/CZ-cl(1) indicated a total of seven synonymous nucleotide differences and seven non-synonymous changes (Table 2). TAHV/68/FR and its biological clone contained identical sequences for each of the segments. TAHV/84/CZ and its biological clone have a total of eight synonymous and two non-synonymous differences in sequence.

**Table 2:** Genetic differences between uncloned and biologically-cloned TAHV

	Nucleotide (amino acid) substitution in indicated segment		
	
Virus	S	M	L
TAHV/58/CZ-cl(2)^a^	No changes	T1073A (F338I)	T673C
		A1097G (N346D)	T2191C
		T1110C (M350T)	T3664C
		G1838A (E593K)	A5152G
		C1846T	T5749A
		T1920C (F630S)	G6533A (D2158N)
		A1954C (K631N)	
		T2995C	
			
TAHV/68/FR-cl^b^	No changes	No changes	No changes
			
TAHV/84/CZ-cl^b^	C729T	A2528G (K830E)	G1520T (D487Y)
		T4468C	A2755G
		C4469T	T4321C
			G6820A
			A6943T
			C6945T

^a ^Genetic comparison between TAHV/58/CZ-cl(1) and TAHV/58/CZ-cl(2).

^b ^Genetic comparison of uncloned parental virus and biologically cloned derivative.

**Figure 1:**
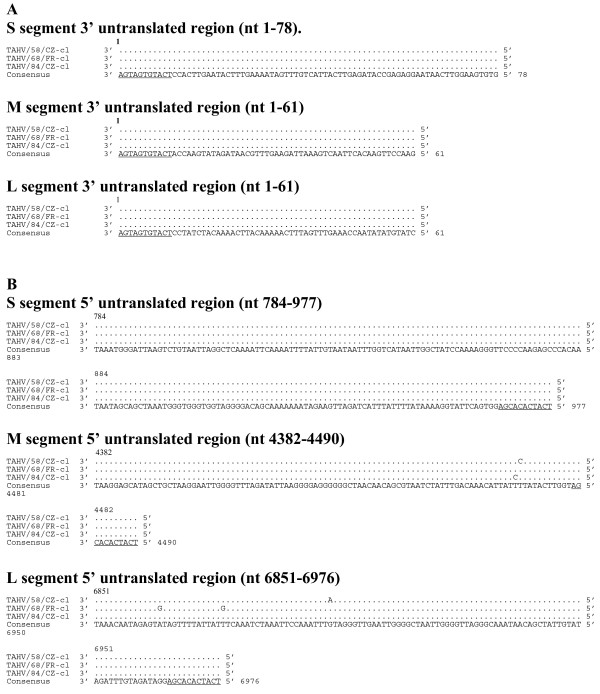
**Alignment of 3' (A) and 5' (B) untranslated regions of the S, M, and L genome segments of TAHV (cDNA presented)**. for each genome segment the consensus sequence consists of two or more sequences sharing the same nucleotide at a given position. Underlined sequence indicates region known to be conserved among orthobunyaviruses. Since differences in the 3' or 5' UTR of TAHV/58/CZ-cl(1) or TAHV/58/CZ-cl(2) were not observed, only one sequence is presented.

The length of the respective S, M, and L genome segments was identical for each TAHV isolate and consisted of 977, 4490, and 6976 nucleotides. Likewise, the length of the ORF for each of the predicted proteins N, NS_S_, M polyprotein, and L was identical in length and contained 235, 97, 1140, and 2263 codons, respectively. TAHV sequence is highly conserved with all viruses sharing greater than 99% nucleotide and amino acid identity. The complete TAHV predicted protein alignment and consensus sequence can be found in Additional File 1 pages 1, 2, and 3. The 3' UTR sequence of the respective S, M, and L segments was identical (Figure 1A). The 5' UTR of each S segment was identical, with the 5' UTR of the M and L segments differing by only two or three nucleotides, respectively (Figure 1B).

### *In vitro *replication kinetics

**Figure 2:**
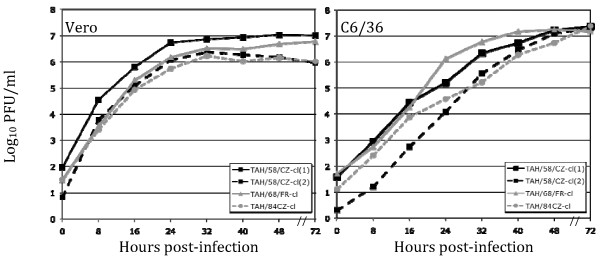
**Growth kinetics of TAHV in Vero and C6/36 cells**. Replication of TAHV/58/CZ-cl (1), TAHV/58/CZ-cl (2), TAHV/84/CZ-cl, and TAHV/68/FR-cl in Vero cells or C6/36 cells infected at an MOI of 0.01. All viruses demonstrated rapid growth with a complete replication cycle in both cell types in less than 8 hours.

The kinetics of *in vitro *replication of TAHV/58/CZ-cl(1), TAHV/58/CZ-cl(2), TAHV/68/FR-cl, TAHV/84/CZ-cl was compared in Vero and C6/36 cells. All TAHV strains exhibit very rapid growth in Vero cells with a full replication cycle in less than eight hours and titers reaching near maximum in 24 hours (Figure 2). In comparison, TAHV grows more slowly in C6/36 cells with titers reaching near maximum in 48 hours (Figure 2). Cytopathic effects (CPE) associated with TAHV infection of Vero cells consisted of cell rounding and detachment from the flask beginning at 24 hours post-infection and complete destruction of the monolayer by 72 hours. CPE was not observed in TAHV-infected C6/36 cells.

### TAHV inoculation of mice

TAHV is highly infectious and neurovirulent for weanling Swiss Webster mice via intracerebral (IC) inoculation with the 50% infectious dose ranging from 0.3 - 1.1 log_10 _PFU, and all viruses cause disease after inoculation (Table 3). The ID_50 _and 50% lethal dose (LD_50_) are identical after IC inoculation indicating very little virus, generally less than 10 PFU, is required to initiate a lethal infection in mice. However, TAHV is 5 to 5,000 fold less infectious for mice following intraperitoneal (IP) inoculation with the ID_50 _ranging from 1.4 - 4.6 log_10 _PFU. TAHV/58/CZ-cl(2), TAHV/68/FR (and clone), and TAHV/84/CZ (and clone) were not neuroinvasive, with LD_50 _levels above 5 log_10 _PFU. TAHV/58/CZ-cl(1) and the uncloned parental stock both manifest a neuroinvasive phenotype not seen with the other TAHV isolates (Table 3). Since we were able to isolate both a neuroinvasive and non-neuroinvasive clone from the same TAHV/58/CZ parental stock, it is unclear which clone represents the authentic wild-type phenotype. Previous work by Janssen *et al. *[21] also demonstrated differences in the neuroinvasive phenotype of TAHV isolates with isolate TAHV/181-57 requiring a very large dose of virus for the development of CNS disease after peripheral inoculation compared to the prototype strain while both viruses maintained a highly neurovirulent phenotype requiring roughly 1 PFU to initiate disease. TAHV/181-57 was passaged 57 times in mouse CNS tissues and was no longer able to replicate in non-neuronal tissues both in vivo and in vitro [21,22]. In the present study, TAHV/58 and THAV/58-cl(2) also appear to be unable to establish an infection after peripheral inoculation as indicated by the low level of seroconversion in the IP inoculated groups (Table 3). TAHV/58/CZ-cl(1) is the only TAHV isolate in this study that was neuroinvasive and was found to contain two amino acid differences from the TAHV consensus sequence (Additional File 1). TAHV/58/CZ-cl(1) differed from the consensus at NS_M _position N346D and at L position D2158N suggesting that one or both of these changes may be responsible for the neuroinvasive phenotype.

**Table 3:** Tahyna virus is highly infectious and neurovirulent for weanling Swiss Webster mice, but strains differ in neuroinvasiveness

	Neuroinvasiveness^a ^(log_10_PFU)		Neurovirulence^b ^(log_10_PFU)		Phenotype^e^	
			
Virus	LD_50_^c^	ID_50_^d^	LD_50_	ID_50_	Neuroinvasive	Neurovirulent
TAHV/58/CZ	3.5	3.5	0.5	0.5	Low	High
TAHV/58/CZ-cl(1)	1.5	1.4	0.7	0.7	High	High
TAHV/58/CZ-cl(2)	>5	4.5	0.8	0.8	Low	High
						
TAHV/68/FR	>5	4.4	0.7	0.7	Low	High
TAHV/68/FR-cl	>5	2.6	0.9	0.9	Low	High
						
TAHV/84/CZ	>5	4.6	1.1	1.1	Low	High
TAHV/84/CZ-cl	>5	3.5	0.3	0.3	Low	High

^a ^Groups of weanling Swiss Webster mice (21-23 days old) were inoculated intraperitoneally with 100 μL of diluted virus (0 - 5 log_10 _PFU).

^b ^Groups of weanling Swiss Webster mice (21-23 days old) were inoculated intracerebrally with 10 μL of diluted virus (1 - 4 log_10 _PFU).

^c ^LD_50 _= Lethal dose 50%.

^d ^ID_50 _= Infectious dose 50%.

^e ^Neuroinvasive phenotype: Low = LD_50 _≥ 3.5 log_10 _PFU; High = LD_50 _≤ 1.5 log_10 _PFU. Neurovirulent phenotype: High = LD_50 _< 1.5 log_10 _PFU.

### Inoculation of rhesus monkeys with TAHV/58/CZ-cl(1)

Subcutaneous infection of rhesus monkeys with TAHV/58/CZ-cl(1) generally elicited a strong neutralizing antibody response in the absence of detectable viremia (Table 4). One monkey (DBXJ) had a serum virus titer of 0.7 log_10 _PFU/mL on day four post-inoculation and also had the highest neutralizing antibody level, indicating that the level of virus replication may correlate with the level of immune response. One monkey (L22) was not infected by the virus. TAHV/58/CZ-cl(1) infection of rhesus monkeys did not result in clinical disease and all animals remained healthy during the study.

**Table 4:** Tahyna virus TAHV/58/CZ-cl(1) is immunogenic in rhesus monkeys in the absence of viremia

Monkey Number	Peak virus titer^a ^(log_10_PFU/mL)	Neutralizing antibody titer on indicated day^b^	
		
		0	42
DBPN	<0.7	<10	2673
DBM6	<0.7	<10	1472
A5E006	<0.7	<10	244
DBXJ	0.7	<10	8379
A5E036	<0.7	<10	1284
L27	<0.7	<10	64
L22	<0.7	<10	<10^d^
DC11	<0.7	<10	701
	**GMT^c^:**	< 10	896

^a ^Serum was collected on days 0, 2, 4, 6, 8, 10, 14, 21, 28, and 42. Viremia was detectable only on day 4 in one monkey. Lower limit of detection is 0.7 log_10_PFU/mL.

^b ^Reciprocal PRNT_60 _against TAHV/58/CZ-cl(1). Lower limit of detection is 10.

^c ^Geometric mean neutralizing antibody titer.

^d ^Monkey considered uninfected and PRNT not used in GMT calculation.

### Sequence comparison of members of the California encephalitis virus (CEV) serogroup

**Figure 3:**
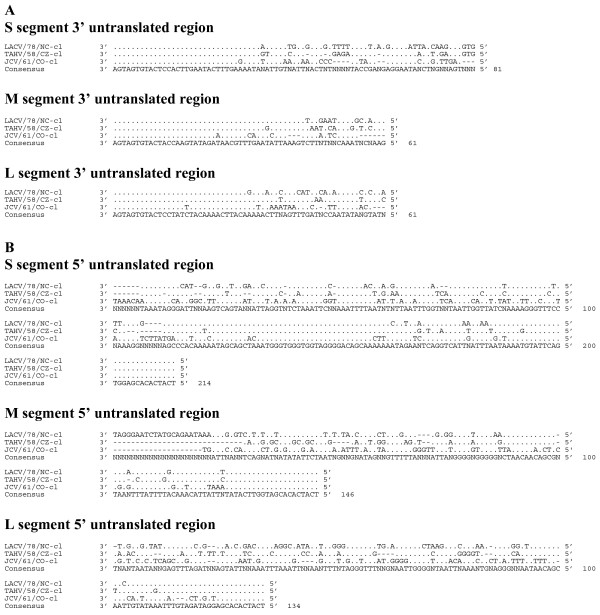
**Alignment of the 3' (A) and 5' (B) untranslated regions of LACV/78-cl, TAHV/58/CZ-cl, and JCV/61/CO-cl**. Sequence identical to the consensus indicated with a (.), areas where no censuses exist indicated by (X), gaps indicated by (-).

Sequence comparison of the nucleotide sequences of the S, M, and L segment ORFs of TAHV, LACV, SSHV, JCV and INKV indicated that these orthobunyaviruses are genetically diverse with 79-84% identity in the S segment, 69-73% identity in the M segment, and 74-78% identity in the L segment compared to TAHV/58/CZ-cl(1) (Table 5). Comparison of the 3' UTR (Figure 3A) and 5' UTR (Figure 3B) of LACV, TAHV, and JCV indicate considerable diversity in both sequence and UTR length and may be a useful target for PCR-based differentiation of the CEV serogroup.

**Table 5:** Nucleotide identity of the orthobunyavirus S, M, and L segment ORFs compared to prototype TAHV/58/CZ-cl(1)

	Percent nucleotide identity		
	
Virus	S	M	L
LACV/78/NC^a^	84	73	77
SSHV/59/MT^b^	84	73	78
JCV/61/CO^c^	79	69	74
INKV/64/FI^d^	80	69	74

^a ^GenBank accession number EF485036 - EF485038.

^b ^GenBank accession number EU294510, EU262553, EU203678.

^c ^GenBank accession number HM007350 - HM007352.

^d ^GenBank accession number U47137, U88059, EU789573.

**Figure 4:**
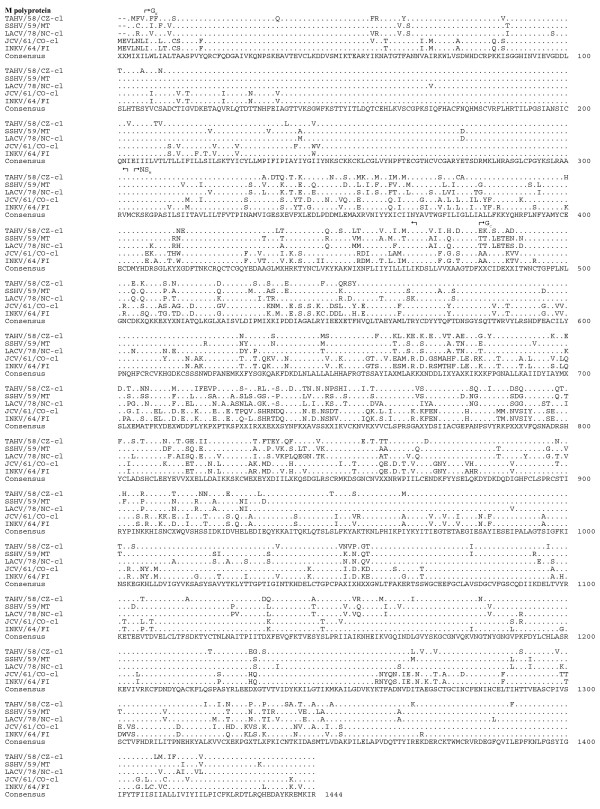
**Alignment of the M polyproteins of TAHV/58/CZ-cl, SSHV/59/MT, LACV/78-cl, JCV/61/CO-cl, and INKV/64/FI**. Sequence identical to the consensus indicated with a (.), areas where no consensus exist indicated by (X).

**Figure 5:**
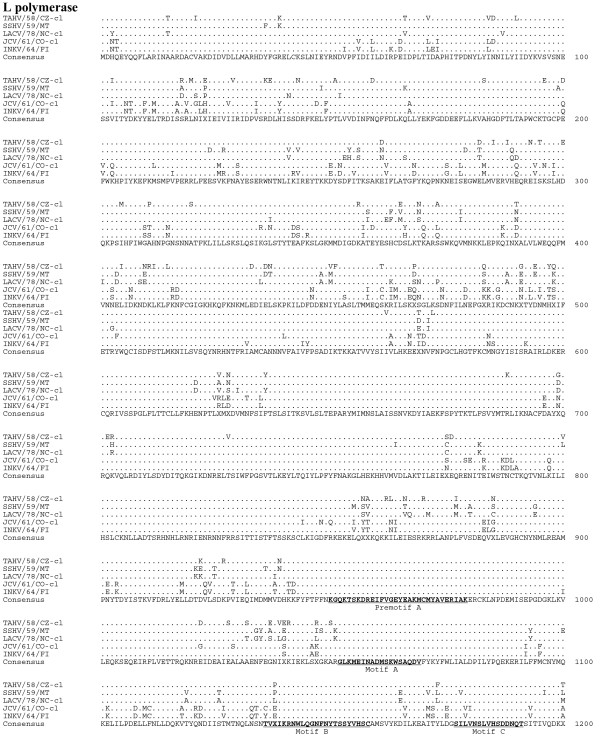
**Alignment of the L proteins (aa 1-1200) of TAHV/58/CZ-cl, SSHV/59/MT, LACV/78-cl, JCV/61/CO-cl, and INKV/64/FI**. Sequence identical to the consensus indicated with a (.), areas where no consensus exist indicated by (X). RNA-dependent RNA polymerase conserved motifs are underlined in the consensus sequence for L protein.

**Figure 6:**
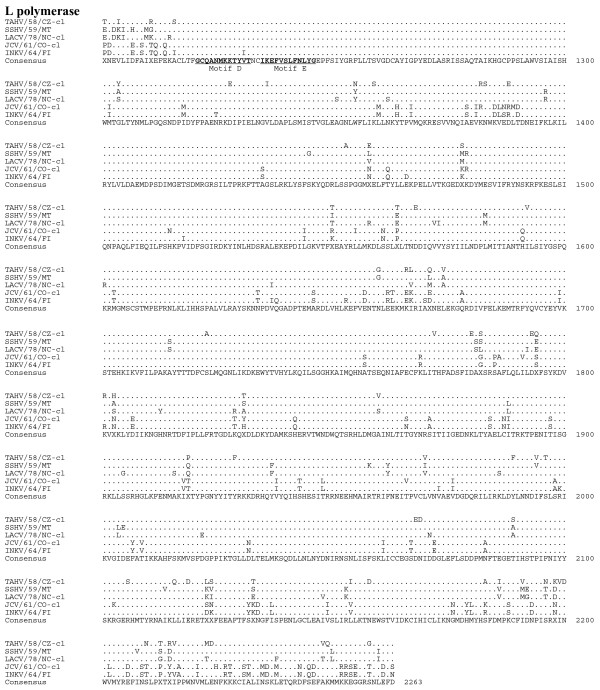
**Alignment of the L proteins (aa 1201-2263) of TAHV/58/CZ-cl, SSHV/59/MT, LACV/78-cl, JCV/61/CO-cl, and INKV/64/FI**. Sequence identical to the consensus indicated with a (.), areas where no consensus exist indicated by (X). RNA-dependent RNA polymerase conserved motifs are underlined in the consensus sequence for L protein.

**Figure 7:**
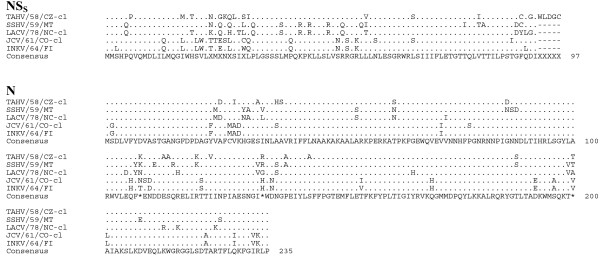
**Alignment of the N and NS_S _proteins of TAHV/58/CZ-cl, SSHV/59/MT, LACV/78-cl, JCV/61/CO-cl, and INKV/64/FI**. Sequence identical to the consensus indicated with a (.), areas where no consensus exist indicated by (X).

Sequence comparison of the predicted amino acid sequences among the serogroup identified the G_N _protein as the most conserved (Table 6, Figure 4). Although the L protein was quite divergent, sharing 83-90% amino acid identity, the six primary structure motifs common to RNA-dependent RNA polymerases were highly conserved, suggesting sequence conservation among functional regions of the protein (Figure 5, 6) [16]. Amino acid identity was lowest among the non-structural proteins, with NS_S _and NS_M _sharing 70-71% identity and 65-75% identity, respectively (Table 6, Figure 7).

**Table 6:** Amino acid identity of the orthobunyavirus predicted protein products compared to TAHV/58/CZ-cl(1)

	Percent amino acid identity with TAHV/58/CZ-cl(1)						
	
Virus^a^	N	NSs	M poly	G_N_	NS_M_	G_C_	L
LACV	86	71	79	93	75	76	89
SSH	88	71	81	92	75	79	90
JCV	84	70	72	88	65	69	83
INKV	85	70	72	87	65	69	82

^a ^GenBank accession numbers shown in Table 5.

### Antigenic relatedness of three CEV serogroup members

To understand the level of antigenic relatedness between CEV serogroup members TAHV, LACV, and JCV, we used rhesus monkey immune serum for cross neutralization analysis. Monkeys were immunized with 10^5 ^PFU biologically-cloned LACV/78/NC-cl, JCV/61/CO-cl, or TAHV/58/CZ-cl(1) and serum was collected 28 days post-inoculation. Each of the three immune sera strongly neutralized the homologous immunizing virus (Table 7). TAHV immune serum weakly neutralized LACV and only minimally neutralized JCV. LACV immune serum weakly neutralized TAHV and did not neutralize JCV. However, JCV immune serum is unique and was capable of cross neutralizing all three viruses (Table 7). Calculations of antigenic relatedness indicate these three viruses, although all members of the CEV serogroup, share less than 6% antigenic relatedness (Table 8). Serological assays based on virus neutralization that are used to identify viruses of the CEV serogroup could be complicated as a result of the broad cross-neutralizing ability of JCV.

**Table 7:** TAHV is antigenically distinct from LACV and JCV by cross neutralizing antibody assay

Immunizing virus^a^		Serum neutralizing antibody titer against indicated virus^b^		
		
	Monkey #	LACV/78/NC-cl	JCV/61/CO-cl	TAHV/58/CZ-cl
LACV/78/NC-cl	DB70	2077	<10	383
	DB8N	222	<10	<10
	CK74	2782	<10	80
	CL2G	326	<10	<10
	DBCZ	4728	<10	169
	CL6E	3719	<10	<10
	DB0H	364	<10	<10
	DA9F	1374	<10	82
	**GMT^c^:**	**1177**	**<10**	**36**
				
JCV/61/CO-cl	A5E054	77	457	<10
	A5E060	2825	352	282
	A5E068	106	221	50
	A5E069	85	712	168
	A5E070	192	662	27
	A5E073	140	256	49
	A5E077	243	685	106
	DBXG	107	407	163
	**GMT**:	**185**	**431**	**69**
				
TAHV/58/CZ-cl	DBPN	92	14	1698
	DBM6	58	<10	494
	A5E006	15	<10	360
	DBXJ	339	31	3195
	A5E036	113	<10	316
	L27	15	<10	215
	DC11	151	<10	1613
	**GMT**:	**68**	**11**	**725**

^a ^Monkeys were immunized subcutaneously with 5 log_10 _PFU of indicated virus and serum was collected on day 28.

^b ^Reciprocal PRNT_60 _assayed against indicated virus. Lower limit of detection is 10.

^b ^Geometric mean titer. For the purpose of calculation, a value of 9 was assigned to monkeys with titers less than 10.

**Table 8:** Percent relatedness between LACV, JCV, and TAHV

	% Relatedness^a^		
	
Viruses compared	LACV/78/NC-cl	JCV/61/CO-cl	TAHV/58/CZ-cl
LACV/78/NC-cl	-	5.7	5.8
JCV/61/CO-cl	5.7	-	4.9
TAHV/58/CZ-cl	5.8	4.9	-

^a ^Percent relatedness = (r_1 _* r_2_)^1/2 ^* 100, where r_1 _is the geometric mean neutralization titer of antibody 1 to homologous strain 1, and r_2 _is the geometric mean neutralization titer of antibody 2 to heterologous strain 1/geometric mean neutralization titer of antibody 2 to homologous strain 2.

## Conclusions

Genetic analysis of three complete TAHV genomes has demonstrated a high level of conservation (99%) at the nucleotide and amino acid level, despite the fact that the viruses were collected from temporally distinct regions spanning a 10 to 26 year period. This level of genetic stability is remarkable and suggests a strong environmental pressure to maintain specific genetic compositions. In nature, the level of genetic stability may be even greater than reported here due to the likelihood that genetic changes may have arisen during *in vitro *passage of the virus following isolation. A similar level of amino acid sequence conservation was also observed with North American LACV, which maintains a more diverse nucleotide sequence (75-87%), but a strict amino acid conservation (90-98%) [23]. We have hypothesized that the unique ecological niche that each CEV serogroup member occupies controls the protein sequence to a greater extent than the nucleotide sequence. This allows for constant drift at the nucleotide level but selection against viruses that are less fit during one or more of the transmission steps. Sequence analysis of additional TAHV strains isolated from multiple insect vectors or amplifying hosts would further elucidate the genetic diversity of TAHV strains.

The virulence phenotype of TAHV is somewhat different in weanling mice than that observed for JCV and LACV. Although all of these CEV serogroup viruses possess a high level of neurovirulence when inoculated directly in brains of mice, JCV and TAHV are generally less neuroinvasive than LACV [24], although a single isolate, TAHV/58/CZ-cl(1), displayed a level of neuroinvasiveness similar to that observed for LACV. The relevance of these mouse phenotypes to the actual encephalitogenic potential in humans is uncertain. When inoculated into non-human primates by a peripheral route, LACV, JCV, and TAHV elicit a robust neutralizing antibody, even in the absence of detectable viremia, as is the case with LACV and TAHV. Inoculation with JCV produced a brief, but significant level of viremia, as high as 10^3 ^PFU/ml in rhesus monkeys [24]. Nevertheless, none of the 24 monkeys inoculated with these viruses displayed any clinical signs of disease. Historically, there is little or no experience with experimental inoculation of non-human primates with the CEV serogroup viruses, so it remains unclear how well these animals mimic the infectious process in humans. However, for humans the sero-prevalence of TAHV is high in endemic areas, but the incidence of clinical disease is remarkably rare. Non-human primates may be accurately reproducing this low incidence of clinical disease, however inoculation of a large number of monkeys would be necessary to observe the infrequent clinical disease if it occurs at all in non-human primates.

We isolated two distinct biological clones from the parental TAHV/58/CZ stock, and these clones differed in their level of neuroinvasiveness. Only two amino acid differences were noted between the neuroinvasive clone, TAHV/58/CZ-cl(1), and the consensus sequence derived from the other TAHV isolates. Individual differences found in the NS_M _and L proteins suggest that one or both of these changes resulted in the ability of this virus to cause CNS disease in mice after peripheral inoculation. We have previously shown for LACV, that a single amino acid change in the G_N _protein abrogated the neuroinvasive phenotype in mice [25]. As an initial step in the development a vaccine against the CEV serogroup, we sought to understand the genetic and antigenic relatedness of TAHV, LACV, and JCV. The attachment glycoprotein G_N _found on the surface of the virion was the most conserved among these orthobunyaviruses suggesting a strongly conserved function, such as tropism for both vertebrate and invertebrate tissues, or the ability of the protein to escape immune selection pressures. Monoclonal antibody raised against the G_N _protein of LACV was able to bind to TAHV and JCV as well, indicating that some epitopes may be shared among these viruses (data not shown). The other attachment glycoprotein, G_C_, was more diverse, but the majority of the diversity was found in the first 400 amino acid residues of N-terminus of the protein. The conservation of these proteins did not translate into cross neutralization for all species. JCV was an exception with infection resulting in a unique broad cross-neutralizing antibody response. However, it has been reported that while mouse antiserum raised against TAHV or Lumbo virus was able to cross-neutralize each virus, serum samples from humans naturally infected with one virus do not reciprocally neutralize the other virus to a significant extent [26]. In the current study, we used primate sera from rhesus monkeys to demonstrate the cross-neutralizing potential of JCV serum, with the expectation that this would more closely mimic the antibody response in humans. Inclusion of the JCV G_N _and G_C _proteins in a CEV serogroup vaccine may result in strong cross protective immune response against viruses found in both North America and Europe, and possibly other regions.

Here we report the first complete genome sequence of TAHV and genetic analysis of complete genomes of new world LACV, SSHV, and JCV with old world TAHV and INKV. As additional virus isolates become available, especially those collected from humans, additional full genome sequences can be determined, and a more extensive analysis can be performed. We have shown using our collection of rhesus immune serum that virus cross neutralization is not uncommon, and this has several important implications. Cross-neutralization may complicate diagnosis and virus identification based on serology, especially in the case of JCV since antibodies elicited by JCV readily cross neutralize both LACV and TAHV. Future diagnostics may need to utilize PCR to differentiate CEV members when virus is available. When only post-infection serum samples are available, multiple viruses of the CEV serogroup will need to be used for neutralization studies. However, the observed cross neutralization also suggests that a single vaccine could generate a cross-neutralizing antibody response which may provide protection against viruses of the CEV serogroup from a wide geographic range. Such vaccine candidates are currently under development in our laboratory.

## Materials and methods

### Cells culture

C6/36 cells (*Aedes albopictus*) were maintained in Earle's MEM supplemented with 10% fetal bovine serum (HyClone, Logan, UT), 2 mM L-glutamine (Invitrogen, Grand Island, NY), and 1 mM non-essential amino acids (Invitrogen). Vero cells (African green monkey kidney) were maintained in OptiPRO™SFM medium (Invitrogen) supplemented with 4 mM L-glutamine.

### Isolation of biologically-cloned viruses

Terminal dilution in Vero cells was used to prepare biological clones TAHV/58/CZ-cl(1), TAHV/68/FR-cl, and TAHV/84/CZ-cl. Virus stocks were serially diluted in 2-fold increments and inoculated onto 90% confluent monolayers of Vero cells in 96-well plates using eight wells per dilution. After five days of incubation at 37°C, cell culture fluid was removed to a holding plate, and the cell monolayers were fixed and stained for 10 minutes with crystal violet solution (1% crystal violet in equal volumes of ethanol and methanol). The virus was selected as a clonal derivative when only 1 or 2 of the 8 wells in a single row was positive for TAHV CPE. Each virus was terminally diluted three times (sequentially), amplified in Vero cell culture, and subjected to genome sequence analysis. TAHV/58/CZ also underwent three rounds of plaque purification and a single round of amplification in Vero cells to generate TAHV/58/CZ-cl(2). For plaque purification, six-well plates with confluent monolayers of Vero cells were infected with serial dilutions of TAHV/58/CZ, and the virus was allowed to attach for one hour. Excess inoculum was removed and cells overlayed with equal volumes of 1.6% SeaPlaque agarose and 2X MEM (Invitrogen) supplemented with 10% FBS and 4 mM glutamine. Plaques were allowed to develop for 4 days, and the wells were overlaid with an additional 2 mL of 1.6% SeaPlaque agarose containing 4% v/v neutral red solution (3.3 g neutral red/L PBS, Sigma Aldrich, St. Louis). After the third plaque pick, virus was amplified in Vero cells.

### RNA Isolation and sequencing

Viral RNA was isolated using either QIAamp Viral RNA kit (Qiagen, Valencia, CA) or High Pure Viral Nucleic Acid Large Volume Kit (Roche, Indianapolis, IN). Overlapping PCR fragments were generated using Titan One Tube RT-PCR Kit (Roche) using TAHV or LACV specific primers. PCR fragments were purified and both strands directly sequenced using viral-specific primers in BigDye-terminator cycle sequencing reactions analyzed on an ABI3730 genetic analyzer (Applied Biosystems, Foster City, CA). Sequence fragments were assembled into a consensus sequence using AutoAssembler 2.1 software (Applied Biosystems).

To sequence the 5' and 3' genome ends of TAHV/58/CZ, viral RNA was isolated using QIAamp Viral RNA kit (Qiagen) from virus infected cells at 24-48 hours post infection for the 3' untranslated region (UTR) or from clarified cell culture fluid for the 5' UTR. Viral RNA was reverse transcribed using Reverse Transcriptor (Roche) at 55°C with random hexamer primers for the 5' UTR or with genome-specific primers for the 3' UTR held at 60-70°C to enhance reverse transcription though RNA secondary structures. cDNA was purified with High Pure PCR product purification kit (Roche) and a poly-A tail was added to the 3' end of the cDNA using 5'/3' RACE Kit, Second Generation (Roche). Genome ends were then amplified using virus and poly-A specific primers. Purified PCR fragments were sequenced as described above.

Once the first and last 13 nucleotides of TAHV/58/CZ were confirmed to be identical to the known consensus sequence for all orthobunyaviruses, primers that had a known sequence abutted to the 13 nucleotide consensus sequence were generated: primer 1(forward) 5'-gaccatctagcgacctccacagtagtgtact- 3' and primer 2 (reverse)

5'-gaccatctagcgacctccacagtagtgtgct-3' (underlined sequence corresponds to genome terminii). These consensus primers were used to determine the 3' and 5' UTR sequence of all remaining TAHV isolates.

### Viral growth kinetics in tissue culture

TAHV/58/CZ-cl(1), TAHV/58/CZ-cl(2), TAHV/68/FR-cl, and TAHV/84/CZ-cl were used to infect 95% confluent monolayers of C6/36 or Vero cells in triplicate, at a multiplicity of infection of 0.01 and incubated for one hour to allow attachment. Infected monolayers were washed three times with sterile PBS and overlaid with medium. Tissue culture supernatant (0.5 mL) was collected at times 0, 8, 16, 24, 32, 40, 48, and 72 hours after infection, mixed with one-tenth volume of 10X SPG buffer (final concentration 218 mM sucrose, 6mM L-glutamic acid, 3.8 mM dibasic potassium phosphate, pH 7.2), and frozen for later titration.

### TAHV clinical disease in mice

The lethal dose_50 _(LD_50_) of TAHV was evaluated in Swiss Webster weanling mice (Taconic Farms, Germantown, NY). All animal experiments were carried out in accordance with the regulations and guidelines of the National Institutes of Health. Twenty-one to twenty-three day-old mice (n = 6/dose) were inoculated with serial dilutions of wildtype or biologically-cloned TAHV in a volume of 10 μL intracerebrally (IC) or 100 μL intraperitoneally (IP). Mice were anesthetized with isofluorane prior to IC inoculation. Following inoculation, all mice were carefully observed twice daily for clinical disease including tremors, seizures, and limb paralysis. Because clinically moribund mice were humanely euthanized before succumbing to infection, moribundity served as a surrogate for the determination of lethality. For determination of the infectious dose_50 _(ID_50_), mice were considered infected if they exhibited clinical disease or developed a detectable serum neutralizing antibody titer.

### Inoculation of rhesus monkeys

Eight sero-negative rhesus monkeys were inoculated subcutaneously with 10^5 ^PFU of biologically-cloned TAHV/58/CZ-cl(1). Serum samples were collected and frozen on days -7, 0, 2, 4, 6, 8, 10, 12, 14, 21, 28, and 42 post inoculation for determination of viremia and neutralizing and cross-neutralizing antibody titer. Monkeys were observed daily for clinical disease.

### Neutralization assay

Neutralizing antibody in mouse and monkey serum was quantified by a plaque reduction neutralization assay. Test sera were heat inactivated (56°C for 30 minutes) and serial 2-fold dilutions beginning at 1:10 were prepared in OptiMEM (Invitrogen) supplemented with 2% FBS, 50 μg/mL gentamicin, and 0.5% human albumin (Talecris Biotherapeutics, Inc., Research Triangle Park, NC). The virus (TAHV, JCV, or LACV) was diluted to a final titer of 500 PFU/mL in the same diluent and 10% guinea pig complement (Cambrex Bioscience Walkersville, Inc., Walkersville, MD) was added to equal volumes of the serum dilutions and mixed well. Serum/virus mixture was incubated at 37°C for 30 minutes, added to confluent monolayers of Vero cells, and incubated for 1 hour to allow virus attachment. Cells were overlayed with 1% methylcellulose and incubated for 5 days at 37°C. To visualize plaques, the overlay was removed, and cell monolayers were washed twice with PBS and immunostained with anti-LACV antibody18752 (QED Bioscience, San Diego, CA) which recognizes JCV, LACV, and TAHV. A 60% plaque-reduction neutralization titer was calculated. Antigenic relatedness was determined using the method of Archetti and Horsfall [27].

## Competing interests

The authors declare that they have no competing interests.

## Authors' contributions

RSB participated in the study design and planning, performed animal studies, data analysis, sequencing analysis, and drafted the manuscript. AKG performed animal studies, sequenced virus isolates, and completed growth curves. BRM and SSW supervised the study and participated in its design and planning. All authors read and approved the final manuscript.

## Supplementary Material

Additional file 1 **TAHV predicted protein alignment**. Sequence identical to the consensus is indicated with a (.), and the areas where no consensus exists are indicated by (X). Unique amino acid differences between neuroinvasive TAHV/58/CZ-cl(1) and the consensus sequence are identified with an arrow (↓). Click here for file
