# Supplementary material for: Tahyna virus genetics, infectivity, and immunogenicity in mice and monkeys
Source: Virol J. 2011 Mar 24;8:135. doi: 10.1186/1743-422X-8-135 (PMC3080826; doi:10.1186/1743-422X-8-135)
Supplement: Additional file 1 — TAHV predicted protein alignment. Sequence identical to the consensus is indicated with a (.), and the areas where no consensus exists are indicated by (X). Unique amino acid differences between neuroinvasive TAHV/58/CZ-cl(1) and the consensus sequence are identified with an arrow (↓). [file 1743-422X-8-135-S1.DOC]

**Page 1**

**NSs**

TAHV/58/CZ-cl(1) .................................................................................................

TAHV/58/CZ-cl(2) .................................................................................................

TAHV/68/FR-cl ..........................................................................................D......

TAHV/84/CZ-cl .................................................................................................

Consensus MMSSPPVQMDLILMQGMWTSVLNMGKQLISIPLGSSSLMPQKPKLLSLVSRRGRLVLNLESGRWRSSIIIFLETGTTQLITTILPSTGCQGIWLDGC 97

**N**

TAHV/58/CZ-cl(1) ....................................................................................................

TAHV/58/CZ-cl(2) ....................................................................................................

TAHV/68/FR-cl ....................................................................................................

TAHV/84/CZ-cl ....................................................................................................

Consensus MSDLVFYDVASTGANGFDPDAGYVDFCIKHGEAINLHSVRIFFLNAAKAKAALARKPERKASPKFGEWQVEVVNNHFPGNRNNPIDNNDLTIHRLSGYLA 100

TAHV/58/CZ-cl(1) ..........D.........................................................................................

TAHV/58/CZ-cl(2) ..........D.........................................................................................

TAHV/68/FR-cl ..........E.........................................................................................

TAHV/84/CZ-cl ..........E.........................................................................................

Consensus RWVLEQFKENXDAAQRELIKTTVINPIAESNGIRWDNGAEIYLAFFPGTEMFLETFNFYPLTIGIYRVKQGMMDPQYLKKALRQRYGSLTADKWMSQKTT 200

TAHV/58/CZ-cl(1) ...................................

TAHV/58/CZ-cl(2) ...................................

TAHV/68/FR-cl ...................................

TAHV/84/CZ-cl ...................................

Consensus AIAKSLKDVEQLKWGRGGLSDTARTFLQKFGIRLP 235

**M polyprotein ⮣G_N_**

TAHV/58/CZ-cl(1) ....................................................................................................

TAHV/58/CZ-cl(2) ....................................................................................................

TAHV/68/FR-cl .......................................................K............................................

TAHV/84/CZ-cl ....................................................................................................

Consensus MMFVLFFIALSAASPVYQRCFQDGAIVKQNPSKQAVTEVCLKDDVSMIKTEARYFRNATGTYANNVAIRKWLVSDWHDCRPKKVSGGHINVIEVGDDLTL 100

TAHV/58/CZ-cl(1) ....................................................................................................

TAHV/58/CZ-cl(2) ....................................................................................................

TAHV/68/FR-cl ....................................................................................................

TAHV/84/CZ-cl ....................................................................................................

Consensus HTEAYVCNADCTIGVDKETAQVRLQTDTTNHFEIAGTTVKSGWFKSTTYITLDQTCEHLKVSCGPKSIQFHACFNQHMSCVRFLHRTILPGSIASSICQN 200

**G_N_** ⮢

TAHV/58/CZ-cl(1) ....................................................................................................

TAHV/58/CZ-cl(2) ....................................................................................................

TAHV/68/FR-cl ....................................................................................................

TAHV/84/CZ-cl ....................................................................................................

Consensus VEIIILTVLTLLIFILLSILSKTYICYILMPIFIPLAYIYGVIYNKSCKKCKLCGLVYHPFTECGTHCVCGARYETSDRMKLHRASGLCPGYKSLRAARV 300

⮣**NS_M_** ↓

TAHV/58/CZ-cl(1) .............................................N......................................................

TAHV/58/CZ-cl(2) .....................................I...........T..................................................

TAHV/68/FR-cl ....................................................................................................

TAHV/84/CZ-cl ....................................................................................................

Consensus MCKSKGPASILSIITAVLILTFVTPINAMVAGDTQETFKLEDLPDDMLSMAMKVNMYYIMCMINYAVTWSFILCALLIALLFKKYQHRFLNFYAMYCHEC 400

**NS_M_⮢ ⮣G_C_**

TAHV/58/CZ-cl(1) ............E.......................................................................................

TAHV/58/CZ-cl(2) ............E.......................................................................................

TAHV/68/FR-cl ............G...................................................E...................................

TAHV/84/CZ-cl ............G.......................................................................................

Consensus DMYHDRSGLKYNXDFTNKCRQCTCGQYEDAAGLLTHRKTYNCLVQYKSKWLTNFLIVYIILMLIKDSVLIVHADGTDFEKCSGEADITWNCTGPFLNLGN 500

TAHV/58/CZ-cl(1) ............................S...............................................................E.......

TAHV/58/CZ-cl(2) ............................S...............................................................K.......

TAHV/68/FR-cl ............................P...............................................................E.......

TAHV/84/CZ-cl ............................P.....................................................L.........K.......

Consensus CEKKQKKESYNNIATQLKGLDAISVLDIXMISKIPEDIAGALRFIEEQRSYHVQLTAEYAMLTRYCDYYTQFTDNSGYSQTTWRVYLRSHDFXACILYPN 600

TAHV/58/CZ-cl(1) ..........................F.........................................................................

TAHV/58/CZ-cl(2) ...................S......F...N.....................................................................

TAHV/68/FR-cl R.........................Y........N.............................................................K..

TAHV/84/CZ-cl ..........................Y.........................................................................

Consensus QHFCRCVKHGDKCSSSNWDFANEMKNXYSGKQSKFDKDLNLALMSLHHAFRGTSSFYIAKLLKEKKNEDLVTYAEKIKGKYPGNALLKAIIDYIKYMEDL 700

TAHV/58/CZ-cl(1) .......Y............................................................................................

TAHV/58/CZ-cl(2) .......Y............................................................................................

TAHV/68/FR-cl .......H....E.............V.........................................................................

TAHV/84/CZ-cl .......H............................................................................................

Consensus TEMNNFKXDEMWDDFIFEVPPTKSPSIRRLESSYDFKTNVNSNPSHICKNIKTVVCLSPKSGVSYDSIIACGEPSQPSIYRKPDSQVFQSNADQTHFCLS 800

TAHV/58/CZ-cl(1) ....................................................................................................

TAHV/58/CZ-cl(2) ....................................................................................................

TAHV/68/FR-cl ......................................................A...........N........E........................

TAHV/84/CZ-cl ......................E.............................................................................

Consensus DTHCLENYETVGEEIIDAIKKSKCWETEFTEYIQFKQSDGVRSCRMKDSGECTVTTNRWPIILCDNDKFYYSELQKDYDKDQDIGHFCLSPRCNTIRHPI 900

TAHV/58/CZ-cl(1) ....................................................................................................

TAHV/58/CZ-cl(2) ....................................................................................................

TAHV/68/FR-cl ....................................................................................................

TAHV/84/CZ-cl ....................................................................................................

Consensus NRKHISNCTWQVSHNNIDKIEVHELEDLEQYKKAITQKLQTSLTLFKYSKTKNLPHIKPMYKYITIEGTETAEGIESAYIESEIPALAGTSIGFKITSKS 1000

TAHV/58/CZ-cl(1) ....................................................................................................

TAHV/58/CZ-cl(2) ....................................................................................................

TAHV/68/FR-cl ....................................................................................................

TAHV/84/CZ-cl ....................................................................................................

Consensus GKHLLDVIGYVKSASYSAVYTKLYTTGPTIGVNTKHDELCTGPCPVNVPHGTGWLTFAKERTSSWGCEEFGCLAISDGCVFGSCQDIIKDEITVYRKETE 1100

Page 2

TAHV/58/CZ-cl(1) ....................................................................................................

TAHV/58/CZ-cl(2) ....................................................................................................

TAHV/68/FR-cl ....................................................................................................

TAHV/84/CZ-cl ....................................................................................................

Consensus ETTDVELCLTFADKTYCTNLNAITPIITDQFEVQFKTVEAYSLPRIIAVRNHEIMVGQINDIGVYSKGCGNVQKVNNTNYGNGVPKFDYLCHLASRKEVI 1200

TAHV/58/CZ-cl(1) ....................................................................................................

TAHV/58/CZ-cl(2) ....................................................................................................

TAHV/68/FR-cl ....................................................................................................

TAHV/84/CZ-cl ....................................................................................................

Consensus VRKCFDNDYQACKFLQTPASYRLEEEGGSVTVIDYKKILGTIKMKAILGDVKYKTFADNVDLTVEGVCTGCINCFENIHCELTIHSTVEASCPVVSSCTV 1300

TAHV/58/CZ-cl(1) ....................................................................................................

TAHV/58/CZ-cl(2) ....................................................................................................

TAHV/68/FR-cl ..........................I.........................................................................

TAHV/84/CZ-cl ....................................................................................................

Consensus FHDRILITPNEHKYAIKVICNEKPGPTLPFKICSAKTDAAMTLADAKPILELAPVDQTTYIKEKDERCKTWMCRVRDEGFQVIFEPFKNLFGSYIGIFYT 1400

**G_C_**⮢

TAHV/58/CZ-cl(1) ........................................

TAHV/58/CZ-cl(2) ........................................

TAHV/68/FR-cl .....................I..................

TAHV/84/CZ-cl ........................................

Consensus FIISLMAIFIVIYIVLPICFKLRDTLRQHEDAYKREMKIR 1440

**L polymerase**

TAHV/58/CZ-cl(1) ....................................................................................................

TAHV/58/CZ-cl(2) ....................................................................................................

TAHV/68/FR-cl .................................................................S..................................

TAHV/84/CZ-cl ....................................................................................................

Consensus MDHQEYQQFLARINTARDACIAKDIDVDLLMARHDYFGKELCKSLNIEYRNDVPFIDIILDIRPETDPLTVDAPHITPDNYLYVDNILYLIDYKVSVSNE 100

TAHV/58/CZ-cl(1) ....................................................................................................

TAHV/58/CZ-cl(2) ....................................................................................................

TAHV/68/FR-cl ....................................................................................................

TAHV/84/CZ-cl ....................................................................................................

Consensus SSIITYDKYYELTRDISRRMNIEIEIVIVRIDPVSKELHISSNRFKELYPALVVDINFDQFFDLKQLLYEKFGDDEEFLLKVSHGDFTLTAPWCKEGCPD 200

TAHV/58/CZ-cl(1) ....................................................................................................

TAHV/58/CZ-cl(2) ....................................................................................................

TAHV/68/FR-cl ....................................................................................................

TAHV/84/CZ-cl ............................................................N.......................................

Consensus FWKHPIYKEFKMSMPVPERRLFEESVKFNAYESERWNTNLIKIREYTKKDYSDFITKSAKDIFLATGFYKQPNKNEISEGWDLMIERIHEQRNITKSLHE 300

TAHV/58/CZ-cl(1) ....................................................................................................

TAHV/58/CZ-cl(2) ....................................................................................................

TAHV/68/FR-cl ....................................................................................................

TAHV/84/CZ-cl ....................................................................................................

Consensus QKPSMHFIWGPHNPGNSNNSTFKLILLSKSLQSIKGLSTYTEAFKSLGKMMDIGDRATEYESHCESLKNKARASWKQVMNKKLEPKQINTALVLWEQQFM 400

TAHV/58/CZ-cl(1) ..........................................................................................G.........

TAHV/58/CZ-cl(2) ..........................................................................................G.........

TAHV/68/FR-cl .........D........................................Y.......................................D.........

TAHV/84/CZ-cl ......................................................................................Y...D.........

Consensus VNNEIIDKNNRIKLLKNFCGIGKHKQFKNKMLDDIDNSKPTILDFDDENVFLASLTMMEQTKRILSKPSGLKSDNFILNEFGQRIKDCNKXTYENMYQIF 500

TAHV/58/CZ-cl(1) ....................................................................................................

TAHV/58/CZ-cl(2) ....................................................................................................

TAHV/68/FR-cl ....................................................................................................

TAHV/84/CZ-cl ....................................................................................................

Consensus ETRYWQCISDFSTLMKNILSVSQYNRHNTFRIAMCANNNVFAIVFPSADIKTKKATVVYSIVVLHKEETNVLNPGCLHGTFKCMNGYISISRAIRLDKER 600

TAHV/58/CZ-cl(1) ....................................................................................................

TAHV/58/CZ-cl(2) ....................................................................................................

TAHV/68/FR-cl ....................................................................................................

TAHV/84/CZ-cl ....................................................................................................

Consensus CQRIVSSPGLFLTTCLLFKHENPTLVMNDVMNFSIYTSLSITKSVLSLTEPARYMIMNSLAISSNVKAYIAEKFSPYTKTLFSVYMTKLIKNACFDAYGQ 700

TAHV/58/CZ-cl(1) ....................................................................................................

TAHV/58/CZ-cl(2) ....................................................................................................

TAHV/68/FR-cl ..K.................................................................................................

TAHV/84/CZ-cl ....................................................................................................

Consensus RERVQLRDIYLSDYDITQKGIKDNRELVSIWFPGSVTLKEYLTQVYLPFYFNAKGLHEKHHVMVDLAKTILEIESDQRENITEIWSTNCTKQTVNLKILV 800

TAHV/58/CZ-cl(1) ....................................................................................................

TAHV/58/CZ-cl(2) ....................................................................................................

TAHV/68/FR-cl ....................................................................................................

TAHV/84/CZ-cl ....................................................................................................

Consensus HSLCKNLLADTSRHNHLRNRIENRNNFRRSITTISTFTSSKSCLKIGDFRKEKELQNAKQKRLLENESRRRRLANPIFVTDEQVNLEVGHCNYNMLREAM 900

TAHV/58/CZ-cl(1) ....................................................................................................

TAHV/58/CZ-cl(2) ....................................................................................................

TAHV/68/FR-cl ....................................................................................................

TAHV/84/CZ-cl ....................................................................................................

Consensus PNYTDYISTKVFDRLYELLDTKVLSDRPVIEQIMDMMVNHKKFYFTFFNKGQKTSKDREIFVGEYEAKMCMYAVERIAKERCKLNPDEMISEPGDGKLKV 1000

TAHV/58/CZ-cl(1) ....................................................................................................

TAHV/58/CZ-cl(2) ....................................................................................................

TAHV/68/FR-cl ..........................................D.........................................................

TAHV/84/CZ-cl ....................................................................................................

Consensus LEQKSEQEIRFLVETTRQKNRDIDEAISALASENFEENVERIEKLSRGKSRGLKMEINADMSKWSAQDVFYKYFWLIALDPILYPQEKERILFFMCNYMQ 1100

Page 3

TAHV/58/CZ-cl(1) ....................................................................................................

TAHV/58/CZ-cl(2) ....................................................................................................

TAHV/68/FR-cl ..................R.................................................................................

TAHV/84/CZ-cl ....................................................................................................

Consensus KELILPDELLFNLLDQKVTYQNDIISTMTNQLNSNTVPIKRNWLQGNFNYTSSYVHSCAMSVYKDILKEAITFLDGSILVNSLVHSDDNQTSITIVQDKT 1200

TAHV/58/CZ-cl(1) .........I..........................................................................................

TAHV/58/CZ-cl(2) .........I..........................................................................................

TAHV/68/FR-cl .........M..........................................................................................

TAHV/84/CZ-cl .........M..........................................................................................

Consensus TNEILIDFAXREFEKSCLTFGCQANMKKTYVTNCIKEFVSLFNLYGEPFSIYGRFLLTSVGDCAYIGPYEDLASRISSAQTAIKHGCPPSLAWVSIAISH 1300

TAHV/58/CZ-cl(1) ....................................................................................................

TAHV/58/CZ-cl(2) ....................................................................................................

TAHV/68/FR-cl ....................................................................................................

TAHV/84/CZ-cl ....................................................................................................

Consensus WMTYLTYNMLPGQSNDPIDYFPAENRKEIPIELNGILDAPLSMISTVGLEAGNLNFLISLLNKYTPVMMKRESVVNQIAEVRSWKVEELTDNEIFKLKIL 1400

TAHV/58/CZ-cl(1) ....................................................................................................

TAHV/58/CZ-cl(2) ....................................................................................................

TAHV/68/FR-cl ...I................................................................................................

TAHV/84/CZ-cl ....................................................................................................

Consensus RFLVLDAEMDPNDIMGETSDMRGRSLLTPRKFTTAGSLRKLYSFSKYQDRLSAPGGMEELFTYLLEKPELLVTKGEDSKDYMESVIFRYNSKRFKESLSI 1500

TAHV/58/CZ-cl(1) ....................................................................................................

TAHV/58/CZ-cl(2) ....................................................................................................

TAHV/68/FR-cl ...S................................................................................................

TAHV/84/CZ-cl ....................................................................................................

Consensus QNPAQLFIEQILFSHKPVIDFSGIRDKYINLHDSRAIEKEPDILGKVTFTEAYRLLMKDLSSLTLTNEDIQVVYSYIILNDPLMITIANTHVLSIYGSPQ 1600

TAHV/58/CZ-cl(1) ....................................................................................................

TAHV/58/CZ-cl(2) ....................................................................................................

TAHV/68/FR-cl ....................................................................................................

TAHV/84/CZ-cl ....................................................................................................

Consensus KRMGMSCSTMPEFRNLKLIHHSPALVLRAYSKNNPDVQGADPTEMARDLVHLKEFVENTGLEEKMRLRIAQNEVEKGQRDIVFELKEMTRFYQVCYEYVK 1700

TAHV/58/CZ-cl(1) ....................................................................................................

TAHV/58/CZ-cl(2) ....................................................................................................

TAHV/68/FR-cl ....................................................................................................

TAHV/84/CZ-cl ....................................................................................................

Consensus STEHKIKVFILPAKAYTTTDFCALMQGNLIKDKEWYTVHYLKQILSGGHKAIMQHNATSEQNIAFECFKLIVHFADSFIEASSRSAFLQLILEQFSYKDV 1800

TAHV/58/CZ-cl(1) ....................................................................................................

TAHV/58/CZ-cl(2) ....................................................................................................

TAHV/68/FR-cl ....................................................................................................

TAHV/84/CZ-cl ....................................................................................................

Consensus RVHKLYDIIKNGHNRTDFIPLLFRTGDLKQTDLDKYDAMKSHERVTWNDWQTSRHLDMGVINLTITGYNRSITIIGEDNKLTYAELCITRKTPENITISG 1900

TAHV/58/CZ-cl(1) ....................................................................................................

TAHV/58/CZ-cl(2) ....................................................................................................

TAHV/68/FR-cl ....................................................................................................

TAHV/84/CZ-cl ..........R.........................................................................................

Consensus RKLLSSRHGLKFENMAKIPTYPGNYYITFRKKDRHQYVYQIHSHESITRRNEEHMAIRTRIFNEITPVCVVNVAEVDGDQRILIRKLDFLNNDVFTLSRI 2000

TAHV/58/CZ-cl(1) ....................................................................................................

TAHV/58/CZ-cl(2) ....................................................................................................

TAHV/68/FR-cl ....................................................................................................

TAHV/84/CZ-cl ....................................................................................................

Consensus KVGIDEFATIKKAHFSKMVSFDGPPIKTGLLDLTELMKSQDLLNLNYDNIRNSNLISFSKLICCEGSEDIDDGLEFLSDDPMNFTEGESIHSTPIFNIYY 2100

↓

TAHV/58/CZ-cl(1) .........................................................D..........................................

TAHV/58/CZ-cl(2) ....................................................................................................

TAHV/68/FR-cl ...............L......S...................................................................I.........

TAHV/84/CZ-cl ....................................................................................................

Consensus SKRGESHMTYRNAIKQLIDRETLSFEEAFTFSTNGFISPENLGCLEAIVSLIRILKTNEWSTVIDKCIHICLIKNGMDHMYHAFDIPKCFVDNPINRKVD 2200

TAHV/58/CZ-cl(1) ...............................................................

TAHV/58/CZ-cl(2) ...............................................................

TAHV/68/FR-cl ..............................................................N

TAHV/84/CZ-cl ...............................................................

Consensus WVMYREFINNLPTTRVPPWNVMMDNFKKKCLALINSKLETQRDFSEFVQMMKKEGGRGNLEFD 2263
